# Sex Differences in Telomere Length in a Bat With Female‐Biased Longevity

**DOI:** 10.1002/ece3.71378

**Published:** 2025-05-14

**Authors:** Jack G. Rayner, Abigail Marshall, Danielle M. Adams, Jillian Kaiser, Katherine Armenta, Gerald S. Wilkinson

**Affiliations:** ^1^ Department of Biology University of Maryland College Park Maryland USA

**Keywords:** ageing, greater spear‐nosed bats, Phyllostomidae, senescence, sexual dimorphism

## Abstract

Telomeres, protective caps at the ends of linear chromosomes, are frequently found to shorten with age. Telomere length is commonly measured in wild populations to investigate age‐related changes in somatic integrity and is considered a hallmark of ageing. Despite interest, there is no clear picture regarding sex differences in telomere length or rate of attrition across species. Bats are of considerable interest in studies of ageing and telomeres, owing to their remarkable longevity and the absence of age‐associated telomere attrition observed in some species. Additionally, multiple bat species show evidence of sex differences in longevity. However, few studies of bat telomeres have included both sexes. We collected DNA from wild‐caught males and females of the highly polygynous greater spear‐nosed bat, *Phyllostomus hastatus* , in which mortality is strongly male‐biased, and measured relative telomere lengths. We found that, while telomeres were shorter in older bats, there was no evidence of shorter telomeres in males. In fact, males tended to have longer telomeres. This runs counter to our prediction of shorter telomeres in the shorter‐lived sex but is not completely unexpected in light of other observations, including that of shorter telomeres in longer lived species.

## Graphical abstract

Despite interest, there is no clear picture regarding sex differences in telomere length across species. We tested for sex differences in telomere length in a bat species in which males live half as long as females. Surprisingly, we found that males appear to have longer telomeres despite their earlier mortality, a pattern that is nevertheless consistent with recent findings in other vertebrate species exhibiting male‐biased sexual size dimorphism.

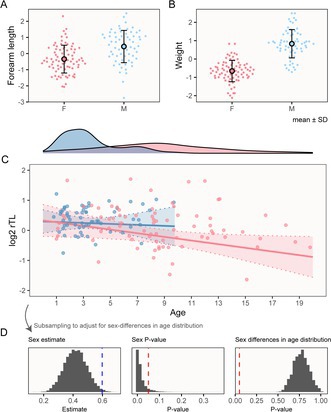

## 1 Introduction

A broad range of animal taxa exhibit sex differences in lifespan, with individuals of one sex consistently outliving the other (Lemaître et al. 2020; Marais et al. 2018). While these differences may ultimately arise from different reproductive strategies of males and females (Tidière et al. 2015), proximate causes of sex differences in longevity are poorly understood (Austad and Fischer 2016) and vary across species, involving a combination of intrinsic and extrinsic factors (Bronikowski et al. 2022). An intrinsic factor of widespread interest in the context of ageing is telomere length. Telomeres are regions of repetitive, non‐coding DNA that cap the ends of linear chromosomes, protecting them against DNA replication errors and oxidative damage, but which shorten during cell division (Aubert and Lansdorp 2008). These regions thus frequently, though not always, shorten with age; telomere attrition is regarded as one of five primary hallmarks of ageing (López‐Otín et al. 2023), or, alternatively, a biomarker of somatic integrity or redundancy (Boonekamp et al. 2013; Wood and Young 2019). Progressive shortening of telomeres is thought to represent a defence against unchecked cell replication associated with tumour growth (Maciejowski and de Lange 2017; Schmutz et al. 2020), but shorter telomeres are also associated with various diseases and disorders and increased mortality risk (Savage and Bertuch 2010; Wilbourn et al. 2018), suggesting a trade‐off that might be influenced by life history strategy.

Across vertebrates, there is a general trend for telomeres to shorten with age, or for older individuals to have shorter telomeres. However, this pattern is variable between species and has notable exceptions (Remot et al. 2022), including species that show evidence of telomere lengthening with age (Tissier et al. 2022). Within species, sex is one likely source of heterogeneity in patterns of telomere attrition, and sex differences in telomere length across animal taxa have been a source of debate. Human populations worldwide exhibit male‐biased mortality (Austad and Fischer 2016), and men typically have shorter telomeres than women (Gardner et al. 2014). Consistent patterns have been remarked upon in other vertebrate taxa, particularly mammals, with authors suggesting males tend to exhibit shorter telomeres or greater rates of age‐associated telomere attrition (Barrett and Richardson 2011). A recent meta‐analysis, however, challenged the view that males tend to have shorter telomeres, finding no evidence of consistent sex differences in telomere length across mammals, or vertebrates more generally, nor any association between sex differences in telomere length or longevity (Remot et al. 2020). This analysis did not test sex differences in the rate of telomere attrition, which could be indicative of differing rates of senescence, and may be a more informative biomarker (Wood and Young 2019), but for which data are comparatively sparse. As noted by the authors, if sexes have similar telomere lengths in early adulthood, but their telomeres shorten at different rates, then sex differences would be most pronounced in older individuals, which are underrepresented in many studies. Additionally, if ages are unknown, and age influences telomere length, comparisons could be confounded by sex differences in the distribution of ages.

Bats are of considerable interest in studies of ageing and telomere length (Power et al. 2022; Teeling 2021). This is owed in part to their impressive longevity relative to similarly sized mammals, with bats having an average body mass‐adjusted lifespan approximately 3.5 times that of other similarly sized placental mammals (Austad 2010; Wilkinson and Adams 2019). Previous studies have suggested maintenance of telomeres might play a role in the extended longevity of some bat species (Foley et al. 2018). Bats also frequently exhibit evidence of sex differences in lifespan, with males or females of different species outliving the other sex, sometimes dramatically so (Adams et al. 2025; Austad 2010). Yet, studies of telomere length in bats have rarely included similar numbers of males and females from across the full range of ages (Foley et al. 2018; Ineson et al. 2020; Power et al. 2022) (but see (Power et al. 2023)), likely due to the difficulty of capturing both sexes in sufficient numbers for many species.

In the current study, we tested whether telomere length is associated with sex or age in Trinidadian populations of the greater spear‐nosed bat *Phyllostomus hastatus* , a highly polygynous species in which males have much earlier mortality. Males of this species compete for and defend harem groups of females (typically with 13–23 females per harem) (McCracken and Bradbury 1981). Perhaps as a result of divergent life histories associated with this harem‐polygynous mating system, males live approximately half as long as females (Adams et al. 2025), which have been reported to live for up to 22 years (Wilkinson and Adams 2019). We extracted DNA from wing biopsies of wild‐caught male and female bats, which we used to measure relative telomere length by qPCR and which were also used in a parallel study to estimate chronological ages from DNA methylation data using a published methylation clock (Wilkinson et al. 2021). We predicted that telomere length would be negatively associated with estimated age and that this negative association would be stronger in males, due to their earlier mortality suggesting potential for accelerated somatic deterioration. By taking advantage of a species with pronounced sex differences in longevity, our study represents an important contribution towards testing predictions of reduced telomere length and greater age‐associated patterns of telomere shortening in the shorter‐lived sex, for which evidence is currently equivocal.

## 2 Methods

### 2.1 Sampling

Tissue samples were collected in January (towards the end of the mating season) of 2023 and 2024. Bats were captured from three locations in Trinidad: natural cave formations in Mount Tamana (10.4711° N, 61.1958° W; *N* = 111 from 2023 and 2024) and the Caura valley (10.7019° N, 61.3614° W; *N* = 28 from 2023), and an abandoned cold storage building in Cumuto (10.5983° N, 61.2117° W; *N* = 22 from 2024). During the day, *P. hastatus* roost in groups of either multiple females (with or without pups) guarded by a single harem male, or multiple ‘bachelor’ or subordinate males. To capture groups, which inhabit small depressions in the cave ceiling, we used a bucket trap, the bottom of which was replaced by a nylon mesh laundry hamper to prevent bats from escaping. This bucket trap was raised to encompass the group, causing bats to fly or drop into the hamper. Bats were individually placed in bags until processing, at which point they were weighed to the nearest 0.1 g, their left forearm measured to the nearest 0.01 mm using digital callipers, and their tooth wear (a correlate of age) scored on a scale of 1 (not worn) to 5 (heavily worn) with 0.5 increments (McCracken and Bradbury 1981). Finally, we took 4 mm wing biopsies from the membrane of each wing of adult bats (Power et al. 2021). Wing membrane biopsies are minimally invasive, and heal quickly (Faure et al. 2009). Tissues were stored in Zymo DNA/RNA shield and frozen at −20°C, until DNA extraction using Zymo Quick‐DNA Miniprep Plus kits. All bats were released back into their roosts. Purified DNA was stored in Zymo DNA Elution Buffer at −20°C. DNA concentrations were measured using high sensitivity Qubit fluorescence assays and were subsequently diluted to 2 ng/μl for use in qPCR reactions. Due to low remaining DNA quantities, we could not systematically assess DNA purity and integrity. In DNA samples extracted under the same conditions, we reliably observe 260/280 ratios ~1.8 on a Nanodrop ND‐1000, and since all samples were collected and extracted using the same protocols any variation in purity should be randomly distributed across samples. We tested DNA integrity using gel electrophoresis for a subset of 26 samples and observed clear bands > 10Kb with minimal smear.

### 2.2 Telomere Length Assays

We estimated relative telomere length (rTL) for each of the samples using qPCR, as in previous studies of rTL using DNA extracted from wing biopsies in bat species (Foley et al. 2018; Ineson et al. 2020; Power et al. 2022, 2023). We used primer sequences *Tel1b* and *Tel2b* (Cawthon 2002) to amplify telomeric sequences, and primers based on the *P. hastatus* genome (Santillán et al. 2021) to amplify brain derived neurotrophic factor *BDNF* (Table S1), which we used as a single‐copy reference gene (see (Foley et al. 2018)). We conducted qPCR assays using PowerUp SYBR green Master Mix for qPCR (Applied Biosystems) on a Roche 480 Lightcycler. Details of qPCR reaction volumes and thermocycler settings are in Table S2. We used dilution series to test amplification efficiencies, which were estimated as 103.66% (95% CI: 96.64%, 111.75%) and 95.63% (95% CI: 87.95%, 104.76%) for telomere and *BDNF* primers, respectively (Figure S1).

We ran each combination of sample and primers in triplicate, with both primers included on the same plate for each of the samples. All plates included a calibrator sample and no‐template control (also run in triplicate). We calculated rTL following Pfaffl (Pfaffl 2001). We ran each plate configuration twice to account for inter‐plate variation and used the mean rTL measurement in our analysis. We ran a subset of samples (*N* = 15) on three plates because the first two rTL estimates were dissimilar. We assessed the range of C*p* values for each triplicate. If the range was greater than 1 and there was a clear outlier, we removed the outlier. Otherwise, we retained the triplicate values on the assumption that the mean is a reasonable estimate but checked that interpretation of results was not affected by the removal of triplicates with a range of values > 1. We performed the same procedure for rTL measures of samples that were run on three plates. Samples for which replicate rTL estimates had coefficients of variation greater than 50% (*N* = 7) were excluded from the analysis, as visualization indicated these were samples with low‐confidence average rTL values. We ran assays in two batches, with the first 86 samples collected in January 2023 and assayed in July/August 2023, and the second 57 samples collected in January 2024 and assayed in May/June 2024. The same reference sample (Band ID 2311) was used in both batches.

Repeatability of log_2_‐transformed primer C*p* values for triplicates and rTL measures from duplicate plates was calculated using rptR (Stoffel et al. 2017), assuming Gaussian error distribution and with 1000 bootstraps. Intraplate repeatabilities of log2‐transformed crossing point values for telomere and BDNF measures were also calculated using rptR, with a covariate of plate ID. Intraplate repeatabilities of crossing point values for telomere and BDNF sequences were 0.933 (95% CI = 0.912, 0.951) and 0.945 (0.925, 0.958), respectively, while interplate repeatability of rTL measures was 0.767 (0.685, 0.827) (Figure S3).

### 2.3 Age Estimation

Methylation data were generated as part of a parallel study (Adams et al. 2025) by submitting purified DNA to the Epigenetic Clock Development Foundation (Torrance, CA), where samples underwent bisulphite conversion and were hybridised to the HorvathMammal40 array. Individual ages were estimated using a published “all bat” clock, designed to accurately estimate chronological age for any bat species based on methylation profiles in skin tissues (Wilkinson et al. 2021). We note that samples from females that appeared older (based on tooth wear or banding records) were sometimes prioritised for methylation profiling in the parallel study, resulting in an underrepresentation of young females in our sample (Adams et al. 2025). This does not apply to males, for which nearly all samples were included in methylation assays. In testing for sex differences in telomere length, we account for this sampling bias by using a subsampling procedure to select for similarly aged males and females, described in detail below.

Among the 143 bats included in the final analysis, 138 had ages estimated by methylation profiles. For the remainder, we knew the ages of two bats based on capture as juveniles, and we estimated the age of four more females based on female‐specific regression of tooth‐wear score on estimated ages (Est. age = 3.0664 × tooth‐wear—0.4828; *R* ^2^ = 0.693, *F*_1,74_ = 166.9, *p* < 0.001) (Wilkinson et al. 2024).

### 2.4 Statistical Analysis

Data were analysed using R (v4.3.1) (Team 2020). Sexual size dimorphism among measures of forearm length and body weight (z‐transformed) was tested using linear regression with predictors of sex and population. For body weight, we included a quadratic effect of estimated age (mean‐centred) using the *poly* function in R, and its interaction with sex, as exploratory plots suggested a non‐linear association that differed between sexes (Figure S2).

The association between mean rTL estimates (averaged across repeated plate runs) per sample and predictor variables of sex, age, forearm length, and population was tested using linear regression, with the response variable log_2_‐transformed to improve the normality of residuals, then z‐transformed (Verhulst 2020). We also included as predictors: year of sampling (2023 or 2024); the interval between sample collection and qPCR assay (Power et al. 2023); and the interval between the date on which the calibrator sample had been collected and qPCR assay. These three predictors were collinear but were included to account for additional confounding variation between rTL measurements (Morrissey and Ruxton 2018). Continuous variables were mean‐centred. We initially included quadratic terms for continuous variables, but removed them as the second‐order terms did not approach significance at *p* < 0.05. Eleven individuals were sampled in both years. Attempts to include ID as a random intercept to account for repeated measures caused singular model fit, so we excluded the January 2023 samples from these individuals in the analysis. Taking the alternative approach of removing the January 2024 samples from our analysis had no effect on the interpretation of results.

We investigated the vulnerability of our results to uncertainty in rTL or age estimates using bootstrap analysis. We retrieved median absolute error (MAE) estimates for estimated age of 0.449 from (Adams et al. 2025) and calculated MAE from our repeated rTL estimates of 0.243. In each of 10,000 bootstraps, we added a random value to age and rTL estimates with a uniform distribution bounded between obs‐AE and obs + MAE, then reran our analysis.

Models with and without interaction terms were tested using type III and II sums of squares, respectively. We used the R package *ggeffects* to plot, from the model of rTL length, adjusted predicted values across sex and ages, controlling for non‐focal terms using ‘empirical’ marginalisation (Lüdecke 2018). Data are available as Supporting Information—S1.

## 3 Results

### 3.1 Body Size

**FIGURE 1:**
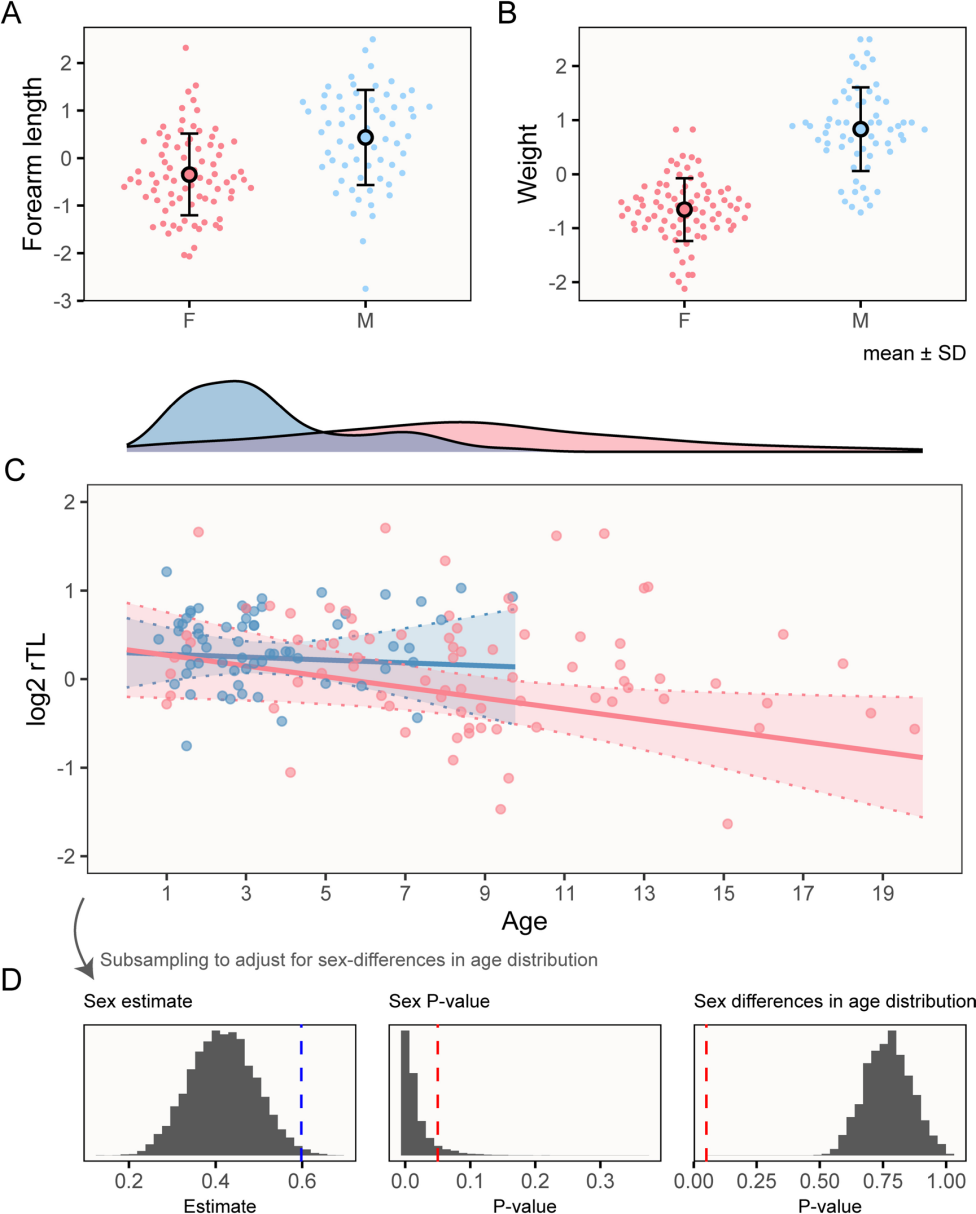
Sexual dimorphism and telomere length. (A, B) show sex differences in measures of forearm length and weight, z‐transformed, illustrating male‐biased sexual size dimorphism in *Phyllostomus hastatus* . Points are distributed along the *x*‐axis according to their density. (C) The relationship between sex, age and log2 rTL (z‐scaled). Points show raw data, lines illustrate predicted values (±95% confidence intervals) from sex‐specific regressions (Table 2) (31). Density curves illustrate sex‐specific age distributions. *N* = 143 samples (80 female, 63 male) in (A–D) Results of the iterative subsampling procedure. Sex estimate and *p*‐value panels show distributions of values from repeating the analysis in Table 2 across 10,000 subsamples in which males and females had similar age ranges. The latter point is illustrated in the far‐right panel, showing *p*‐values from Wilcoxon rank‐sum tests against a null hypothesis of equal age distribution. Red dashed lines illustrate *p*‐value significance thresholds of 0.05, whereas the blue dashed line illustrates the observed sex effect from Table 2.

We observed substantial sexual size dimorphism, consistent with previous findings (Adams et al. 2020), and with predictions for highly polygynous mating systems (Clutton‐Brock 1985). Males had larger structural body size (Figure 1A) and were much heavier (Figure 1B) across ages. Body weight was also associated with a quadratic term of estimated age, which differed between the sexes (Table 1, Figure S2). Distributions of estimated ages differed substantially between the sexes (Figure 1C), with a maximum female age of 19.80 years (median = 8.30, IQR [5.70, 11.25]), versus 9.70 years for males (median = 3.00, IQR [1.80, 4.20]) (Figure 1C). Samples were non‐random with respect to female age distributions (see Methods), so we did not statistically compare estimated age distributions in our sample. Nevertheless, the sex difference in maximum age is consistent with observations of female‐biased longevity in this species (Adams et al. 2025; Wilkinson et al. 2024) and we sampled across the full range of adult ages in both sexes.

**TABLE 1:** Results of regressions of z‐scaled measures of forearm length (*F*_3,139_ = 13.72, *p* < 0.001, *R* ^2^ = 0.227) and body weight (*F*_7,135_ = 42.15, *p* < 0.001, *R* ^2^ = 0.667) against predictors of sex and population (*N* = 143).

Response	Predictor	Coef. ± SE	*F*	df	*p*
Forearm length	Intercept	−0.500 ± 0.189			
	Sex (M)	0.860 ± 0.152	31.951	1	< 0.001
	Population		6.806	2	0.002
	*Caura*	−0.290 ± 0.193			
	*Cumuto*	−0.800 ± 0.220			
	Residuals	± 0.888		139	
Body weight	Intercept	−0.831 ± 0.081			
	Sex (M)	1.896 ± 0.282	45.274	1	< 0.001
	Population		3.654	2	0.031
	*Caura*	0.328 ± 0.128			
	*Cumuto*	0.174 ± 0.143			
	*Est.age*		3.554	2	0.035
	Est.age	2.021 ± 0.843			
	Est.age^2^	−1.376 ± 0.720			
	Sex × Est.age^2^		6.282	2	0.002
	*SexM:Est.age*	2.558 ± 5.960			
	*SexM:Est.age* ^2^	−4.171 ± 4.193			
	Residuals	± 0.577		135	

### 3.2 Relative Telomere Length

As predicted, telomere length (averaged across replicate plates) was negatively associated with age in the full model, including both sexes, of our cross‐sectional sample (Table 2). Surprisingly, however, telomeres of males were estimated to be longer despite their earlier mortality. Given different age distributions in males and females, we did not fit a sex by age interaction in the full model but ran sex‐specific models which reported a significant negative association between age and telomere length only across female samples, perhaps influenced by the larger sample size (*N* = 80, to 63 for males) and wider range of ages (as females live up to twice as long as males) (Table 2).

**TABLE 2:** Results of linear regression of log_2_ mean rTL, z‐transformed.

	Predictor	Coef. ± SE	*F*	df	*p*
Full model (*N* = 143)	Intercept	11.323 ± 5.039			
	Forearm length	−0.060 ± 0.048	1.568	1	0.213
	Est. age	−0.054 ± 0.022	5.981	1	0.016
	Sex (M)	0.491 ± 0.205	5.705	1	0.018
	Population		0.983	2	0.377
	*Caura*	−0.311 ± 0.265			
	*Cumuto*	−0.242 ± 0.285			
	Year sampled (Jan_2024)^a^	−29.492 ± 12.588	5.488	1	0.021
	Sample storage duration^a^	−0.098 ± 0.035	7.879	1	0.006
	Calibrator sample storage duration^a^	0.081 ± 0.035	5.281	1	0.023
	Residuals	± 0.896		133	
Females (*N* = 80)	Intercept	12.143 ± 10.126			
	Forearm length	−0.048 ± 0.078	0.390	1	0.534
	Est. age	−0.061 ± 0.028	4.737	1	0.033
	Population		1.033	2	0.361
	*Caura*	−0.535 ± 0.405			
	*Cumuto*	0.215 ± 0.467			
	Year sampled (Jan_2024)^a^	−25.839 ± 21.248	1.479	1	0.228
	Sample storage duration^a^	−0.096 ± 0.058	2.756	1	0.101
	Calibrator sample storage duration^a^	0.068 ± 0.060	1.307	1	0.257
	Residuals	± 1.043		71	
Males (*N* = 63)	Intercept	6.666 ± 4.408			
	Forearm length	−0.054 ± 0.058	1.304	1	0.355
	Est. age	−0.016 ± 0.050	0.061	1	0.747
	Population		1.584	2	0.304
	*Caura*	0.021 ± 0.322			
	*Cumuto*	−0.541 ± 0.348			
	Year sampled (Jan_2024)^a^	−21.015 ± 14.419	1.778	1	0.151
	Sample storage duration^a^	−0.065 ± 0.041	2.197	1	0.114
	Calibrator sample storage duration^a^	0.060 ± 0.040	1.862	1	0.144
	Residuals	± 0.680		55	

*Note:* The full model had an *R* ^2^ of 0.243 (*F*_8,133_ = 5.336, *p* < 0.001). Female‐ and male‐only models had *R* ^2^ values of 0.254 (*F*_7,72_ = 3.453, *p* = 0.003) and 0.178 (*F*_7,55_ = 1.702, *p* = 0.073).

^a^ These terms are collinear and are included to account for confounding variation between samples, but their corresponding estimates should not be interpreted individually (see Tables S5–S7).

A potential issue with the interpretation that males have longer telomeres, based on the significance of the sex term in the full model, is that there are clear differences in age distributions between males and females in our sample (Figure 1C) which could confound their comparison. To investigate whether the observed sex difference in rTL was an artefact of the different age distributions, we performed an iterative subsampling procedure to test for sex differences in relative telomere length across random subsamples in which the sexes had similar age distributions. Briefly, we retained, for each male in our sample, a single female within 1 year of age (if there was one, otherwise the male was discarded from the sample). Males were reported to have significantly (*p* < 0.05) longer telomeres in 91.99% of 10,000 random samples, supporting the initial interpretation, though the magnitude of the difference was reduced compared with the full model (Figure 1D). In sum, we did not observe clear support for predictions of accelerated telomere attrition or shorter telomeres in relatively short‐lived male *P. hastatus* , and our results are directly contrary to the latter prediction.

Neither the association with sex nor age, reported by the full model, appeared to be influenced by uncertainty in rTL measures or estimated ages (Figure S5). We did observe significant effects of variation in sample and calibrator storage duration, which were collinear and differed between years (Table 2). Removal of collinear terms did not influence biological interpretation (Tables S3–S5). Because of this technical variation, we could not directly compare rTL measures for 11 bats sampled in both 2023 and 2024, but comparison of regression residuals between years indicated no clear direction of change (i.e., consistent gain or loss of rTL) (Figure S4).

## 4 Discussion

Sex is an important source of variation in rates of mortality and senescence in wild animal populations (Bronikowski et al. 2022; Marais et al. 2018), and telomere attrition is regarded by some as a hallmark of ageing or senescence (López‐Otín et al. 2023). Whether sexes differ in telomere lengths, constitutively or via divergent life histories, is unclear. Like many bat species, *P. hastatus* has a longer maximum lifespan than predicted for a mammal of its size (Wilkinson and Adams 2019), yet males live approximately half as long as females. As previously reported, males are also larger (McCracken and Bradbury 1981), with size likely an important factor influencing their ability to compete for and defend large groups of females, and potentially also their relatively short lifespan (Clutton‐Brock et al. 1985). While we observed significant age‐related variation in telomere length across our sample, we did not observe longer telomeres in females and, in fact, this pattern appeared to be reversed, with males tending to have longer telomeres.

Various factors have been proposed to contribute to the apparent trend for male mammals to exhibit shorter telomeres (Barrett and Richardson 2011), which could be reversed in some species. Heterogametic males might be disadvantaged if deleterious mutations affect X‐linked genes involved in telomere maintenance, such as *DKC1* in humans (Barrett and Richardson 2011). This pattern might be reversed in taxa such as birds and lepidoptera, in which females are the heterogametic sex (Horn et al. 2011). *P. hastatus* males are heterogametic, so this could not explain the pattern we observe, and this prediction was also not supported by a meta‐analysis of sex differences in telomere length (Remot et al. 2020). Alternatively, variation in telomere length could arise as a consequence of sexual size dimorphism. Among mammals exhibiting sexual size dimorphism, males tend to be the larger sex, but this is not always the case (Tombak et al. 2024). Investment in growth and maintenance of larger body mass are associated with increased cell replication which, in the absence of telomerase activity, is expected to reduce telomere length (Barrett and Richardson 2011). Male *P. hastatus* are larger both in skeletal size and body mass but have longer telomeres, so this prediction also does not explain our findings.

Our observation that male *P. hastatus* tended to have longer telomeres, despite larger body size and earlier mortality, was counter to our prediction. Sex differences in telomere length could be constitutive or might arise if sexes differentially invest in telomere maintenance, for example, via telomerase enzymes, which have recently been found to be expressed in bat wing tissues (Athar et al. 2024; Li et al. 2023; Power et al. 2023) and expression of which is correlated with mass across mammals (Gomes et al. 2011). Our finding of longer telomeres in males of a bat with male‐biased mortality appears consistent with the view that the pattern of shorter telomeres in longer‐lived species (Gomes et al. 2011), perhaps owing to the role of telomere degradation in tumour defence, might be recapitulated at the within‐species level (Tissier et al. 2022). Relatively short‐lived males of *P. hastatus* might be less exposed to selection on tumour defences due to earlier mortality, which could favour selection against adverse early‐life effects of shorter telomeres (Wilbourn et al. 2018). On the other hand, the inverse association between species mass and telomere length (Pepke and Eisenberg 2022) would, if applied at the within‐species level, predict that larger males would have shorter telomeres. In the case of *P. hastatus* , such an effect might be counteracted by strongly accelerated male mortality. However, on this point, our observations are also consistent with a finding of a recent meta‐analysis finding that among vertebrates with male‐biased sexual size dimorphism, males tended to have longer telomeres (Remot et al. 2020). Remot et al. were evidently unconvinced by this finding and found that removal of a mandrill ( *Mandrillus sphinx* ) dataset (Beaulieu et al. 2017) rendered this pattern non‐significant, but we think it might warrant further consideration.

We note certain caveats to be considered when interpreting our results. As in other cross‐sectional studies, it is possible that the patterns we observe are influenced by the selective disappearance of individuals with shorter telomeres. Moreover, selection on telomere length could differ between the sexes (Bauch et al. 2020), and could therefore contribute to observed differences in telomere length both across ages and between sexes. For example, male‐biased selective disappearance of bats with shorter telomeres, perhaps associated with the strongly polygynous mating system, could conceal a pattern of telomeric attrition in males of our cross‐sectional sample and suggest that males tend to have longer telomeres among age‐matched individuals. Next, the distribution of ages in our sample differs between the sexes. With respect to the range of ages, this is biologically representative (Adams et al. 2025; Wilkinson et al. 2024), and we found that restricting our analysis to an unbiased subsample did not affect the interpretation of longer telomeres in males. Finally, we found that telomere length measurements were influenced by sample storage duration. This was unexpected, as a previous study using qPCR to measure bat telomeres observed no effect of storage duration under similar conditions over a longer duration (Power et al. 2023), but the inclusion of these factors in our model should account for this technical variation.

Given widespread interest in the factors underlying extended longevity in bats (Gorbunova et al. 2020; Teeling 2021), there have been relatively few studies of telomere length. Among the few published studies, there is mixed evidence of age‐associated declines across taxa (Foley et al. 2018, 2020; Forest 2022; Ineson et al. 2020; Power et al. 2023). Our study adds to the phylogenetic diversity represented for this clade, as the only representative of the *Phyllostomidae*, one of the most ecologically diverse mammalian groups (Leiser‐Miller and Santana 2020). Patterns of age‐associated telomere attrition are variable across bat species, with species of the genus *Myotis* appearing not to show evidence of attrition (Foley et al. 2018; Ineson et al. 2020). However, with our findings taken into account, there does appear to be a general trend for age‐related decline in telomere length in bat species (Foley et al. 2018; Forest 2022; Power et al. 2023), suggesting that telomere maintenance is not a distinguishing feature underlying their extraordinary longevity.

## Author Contributions

**Jack G. Rayner:** data curation (equal), formal analysis (lead), investigation (equal), project administration (lead), writing – original draft (lead), writing – review and editing (lead). **Abigail Marshall:** data curation (equal), investigation (equal), writing – review and editing (supporting). **Danielle M. Adams:** investigation (supporting), writing – review and editing (supporting). **Jillian Kaiser:** investigation (supporting), writing – review and editing (equal). **Katherine Armenta:** investigation (supporting), writing – review and editing (supporting). **Gerald S. Wilkinson:** data curation (equal), funding acquisition (lead), investigation (equal), resources (lead), writing – review and editing (equal).

## Conflicts of Interest

The authors declare no conflicts of interest.

## Supporting information


**Appendix S1.** Supporting Information.

**Appendix S2.** Supporting Information.

**Appendix S3.** Supporting Information.

## Data Availability

Data and associated scripts underlying our results are included as supporting information—S1.
