# Supplementary material for: Sex Differences in Telomere Length in a Bat With Female‐Biased Longevity
Source: Ecol Evol. 2025 May 14;15(5):e71378. doi: 10.1002/ece3.71378 (PMC12076057; doi:10.1002/ece3.71378)
Supplement: Supplementary file 2 — Appendix S2. Supporting Information. [file ECE3-15-e71378-s002.docx]

####

# Sex differences in telomere length in a bat with female-biased longevity

# Jack G. Rayner, Abigail Marshall, Danielle M. Adams, Jillian Kaiser, Katherine Armenta, Gerald S. Wilkinson

####

library(ggplot2)

library(car)

library(ggbeeswarm)

library(patchwork)

library(ggeffects)

library(ggExtra)

library(ggforce)

library(viridis)

library(rptR)

#plot efficiency

eff<-read.csv('./primer_efficiency_all.csv',h=T)

summary(lm(Cp~log10(dilution),dat=eff[eff$primers=="Tel",]))#95% CI slope = -3.23714 (-3.40514,-3.06914)

summary(lm(Cp~log10(dilution),dat=eff[eff$primers=="BDNF",]))#95% CI slope = -3.23714 (-3.649,-3.213)

effplot<-ggplot(eff,aes(x=log10(dilution),y=Cp))+

geom_point(alpha=0.25)+stat_summary(fun='mean')+geom_smooth(method='lm',colour='black')+

facet_wrap(.~primers,scales='free')+

theme_bw()+

labs(subtitle="Tel slope = -3.237 Â± 0.084 SE, amplification factor = 2.04, efficiency = 103.66%\nBDNF slope = -3.431 Â± 0.109 SE, amplification factor = 1.96, efficiency = 95.63%")

#ggsave('efficiency_plot.png',plot=effplot,dpi=600,height=4,width=6)

#read in data

dat<-read.csv('Filtered_EcoEvo_R1_Supporting_Information_Ph_rTLdata.csv',h=T)

dat$sex<-factor(dat$sex)

#remove jan_23 vals for duplicate bands

dupes<-dat[duplicated(dat$band),]$band

dupes.df<-dat[dat$band %in% dupes,]

dupes.df$band<-factor(dupes.df$band)

dat<-dat[!(dat$band %in% dupes.df$band & dat$Season_sampled=="Jan_23"),]

#does it make a difference if we instead remove the jan_24 duplicate band values? - No

#dat<-dat[!duplicated(dat$band),]

#estimate ages of unaged samples (all female) based on toothwear scores

summary(lm(Est.Age~TTH,data=dat[dat$sex=="F" & !dat$AgeSource=="ToothWear",]))

ggplot(dat,aes(x=TTH,y=Est.Age,colour=sex))+scale_colour_manual(values=c("#fa8490","#9fd4fc"))+

theme_minimal()+geom_point()+geom_smooth(method='lm')

dat[dat$AgeSource=="ToothWear" & dat$sex=="F",]$Est.Age<-

dat[dat$AgeSource=="ToothWear" & dat$sex=="F",]$TTH*3.0664-0.4828

#test if removing samples with multiple flags affects interpretation

#dat <- dat %>%

# filter(rowSums(across(starts_with("FLAG"), ~ . == "FLAG")) < 1)

#test interplate repeatability of rTL

dat<-dat[!dat$tlCV>0.5 | dat$band=="2311",]

#samples with rtl CV>0.5 are not reliable

ggplot(dat1[dat1$rep %in% c("rep1","rep2"),],aes(x=rep,y=log2(rtl)))+geom_line(aes(group=band,colour=tlCV),alpha=0.5,linewidth=1)+

theme_minimal()+theme(panel.grid=element_blank())

dat1<-data.frame(band=rep(dat$band,3),tlCV=rep(dat$tlCV,3),tlSTDEV=rep(dat$rtl_STDEV,3),rtl=c(dat$rtl1,dat$rtl2,dat$rtl3),rep=c(rep("rep1",nrow(dat)),rep("rep2",nrow(dat)),rep("rep3",nrow(dat))),MonthAssay=dat$MonthAssay,Season_sampled=dat$Season_sampled)

rpt.rtl<-rpt(log2(rtl) ~ (1|band), grname = "band", data = dat1, datatype = "Gaussian",

nboot = 1000, npermut = 0)

#test intraplate repeatability of tel primers

dat1<-data.frame(band=rep(dat$band,3),tel=c(dat$tel1_a,dat$tel1_b,dat$tel1_c),plate=dat$plate)

rpt.tel<-rpt(log2(tel) ~ plate+(1|band), grname = "band", data = dat1, datatype = "Gaussian",

nboot = 1000, npermut = 0)

#test intraplate repeatability of BDNF primers

dat1<-data.frame(band=rep(dat$band,3),bdnf=c(dat$bdnf1_a,dat$bdnf1_b,dat$bdnf1_c),plate=dat$plate)

rtl.bdnf<-rpt(log2(bdnf) ~ plate+(1|band), grname = "band", data = dat1, datatype = "Gaussian",

nboot = 1000, npermut = 0)

#plot repeatabilities

png('repeatabilities.png',res=600,width = 4,height=6,unit='in')

par(mfrow=c(3,1),mar=c(2,2,2,2))

plot(rpt.tel,xlim=c(0,1),main="intraplate repeatability (Tel)")

plot(rtl.bdnf,xlim=c(0,1),main="intraplate repeatability (BDNF)")

plot(rpt.rtl,xlim=c(0,1),main="interplate repeatability (rTL)")

dev.off()

#sex-specific age summary

summary(dat[dat$sex=="F",]$Est.Age)

summary(dat[dat$sex=="M",]$Est.Age)

summary(factor(dat$sex))

#run forearm/Weight regressions

dat$population<-relevel(factor(dat$population),ref="Tamana")

lm.fa<-lm(scale(FA)~sex+population,data=dat)

lm.wt<-lm(scale(WT)~sex+population+sex*poly(scale(Est.Age,scale=FALSE),2),data=dat)

Anova(lm.fa,type="II")

Anova(lm.wt,type="III")

summary(lm.fa)

summary(lm.wt)

#plot(lm.fa)#good

#plot(lm.wt)#good

#plot size and age across sexes

g.fa<-ggplot(dat,aes(x=sex,y=scale(FA),colour=sex))+

scale_colour_manual(values=c("#fa8490","#9fd4fc"))+

scale_fill_manual(values=c("#fa8490","#9fd4fc"))+

geom_quasirandom(size=0.57)+

stat_summary(fun.data=mean_sdl,fun.args=list(mult=1),

colour='black',geom="errorbar",width=0.1)+

stat_summary(fun="mean",shape=21,colour='black',aes(fill=sex))+

theme_minimal()+theme(legend.position='none',axis.title.x=element_blank())+

ylab("Forearm length")+

theme(panel.grid=element_blank(),axis.ticks=element_line(),panel.background=element_rect(fill='#fcfbfa',colour='black'))+

labs(tag="A",caption=" ")

g.wt<-ggplot(dat,aes(x=sex,y=scale(WT),colour=sex))+

scale_colour_manual(values=c("#fa8490","#9fd4fc"))+

scale_fill_manual(values=c("#fa8490","#9fd4fc"))+

geom_quasirandom(size=0.57)+

stat_summary(fun.data=mean_sdl,fun.args=list(mult=1),

colour='black',geom="errorbar",width=0.1)+

stat_summary(fun="mean",shape=21,colour='black',aes(fill=sex))+

theme_minimal()+theme(legend.position='none',axis.title.x=element_blank())+

ylab("Weight")+

theme(panel.grid=element_blank(),axis.ticks=element_line(),panel.background=element_rect(fill='#fcfbfa',colour='black'))+

labs(tag="B",caption="mean Â± SD")

#exploratory plot showing non-linear assocation between age and weight

g.wt.nonlinear<-ggplot(dat,aes(x=Est.Age,y=scale(WT),colour=sex))+

scale_colour_manual(values=c("#fa8490","#9fd4fc"))+

scale_fill_manual(values=c("#fa8490","#9fd4fc"))+

geom_point(size=0.57)+

geom_smooth(method='loess',span=1.5)+

theme_minimal()+theme(legend.position='none',axis.title.x=element_blank())+

ylab("Weight")+xlab("Est. Age")+

theme(panel.grid=element_blank(),panel.background=element_rect(fill='#fcfbfa',colour='#aaaaaa'))

#ggsave('age_weight_loss.png',plot=g.wt.nonlinear,dpi=600,height=4,width=5.5)

#run rTL model

dat$log2tlmean<-log2(dat$tlmean)

rtl.lm<-lm(scale(log2tlmean)~scale(FA,scale=FALSE)+scale(Est.Age,scale=FALSE)+

sex+population+#Season_sampled+

scale(days_collection_to_assay,scale=FALSE),#+

# scale(days_since_calibrator_extracted,scale=FALSE),

#data=dat[dat$Est.Age<8,])

data=dat)

#plot(rtl.lm)#looks fine

summary(rtl.lm)

Anova(rtl.lm,type="II")

#check residual rTL of repeated measure bats (only if duplicates haven't been removed)

# dat$residRTL<-resid(rtl.lm)

# dupes<-dat[duplicated(dat$band),]$band

# dupes.df<-dat[dat$band %in% dupes,]

# resid.rtl.year<-ggplot(dat,aes(x=Season_sampled,y=residRTL,colour=sex))+

# theme_bw()+geom_quasirandom(alpha=0.5)+

# geom_point(data=dupes.df,aes(x=Season_sampled,y=residRTL,colour=sex))+

# theme(panel.grid=element_blank(),panel.background=element_rect(fill='#fcfbfa',colour='#aaaaaa'))+

# geom_line(aes(group=band))+scale_colour_manual(values=c("#fa8490","#9fd4fc"))

# ggsave('residual_rtl_year.png',plot=resid.rtl.year,dpi=600,height=4,width=4)

# plot adjusted predicted values of rTL across sexes and ages, based on rtl.lm model

# empirical marginilisation appears to be recommended when you have skewed non-focal variables,

# which we do (e.g., sig difference in number of Jan_23 and Jan_24 samples)

g <- predict_response(rtl.lm, terms=c("Est.Age","sex"),back_transform = FALSE,margin='empirical',ci_level = 0.95)

g1<-data.frame(g)

#plot rTL across ages with predicted values from regression

g.rtl<-ggplot(dat,aes(x=Est.Age,y=log2tlmean,colour=sex),) +

geom_line(data=g1[g1$group=="M",],aes(x=x,y=predicted),colour="#5090bf",size=1) +

geom_ribbon(data=g1[g1$group=="M",],linetype='dotted',

aes(x=x,y=predicted,ymin=conf.low,ymax=conf.high),alpha=0.2,colour="#5090bf",fill="#5090bf")+

geom_rect(fill='#fcfbfa',aes(xmin=9.8,xmax=20,ymin=-2,ymax=2),colour='#fcfbfa')+

geom_line(data=g1[g1$group=="F",],aes(x=x,y=predicted),colour="#fa8490",size=1) +

geom_ribbon(data=g1[g1$group=="F",],linetype='dotted',

aes(x=x,y=predicted,ymin=conf.low,ymax=conf.high),alpha=0.2,colour="#fa8490",fill="#fa8490")+

geom_point(alpha=0.6)+

scale_x_continuous(breaks=seq(1,20,2))+labs(tag="C")+

labs(x = "Age",y = "log2 rTL",color = "Sex") +

theme_bw()+scale_colour_manual(values=c("#fa8490","#5090bf"))+

theme(panel.grid=element_blank(),legend.position='none',panel.background=element_rect(fill='#fcfbfa',colour='#aaaaaa'))

g.rtl

ggsave('fig1_size_rtl.tif',dpi=600,height=7.5,width=6,

plot=(g.fa | g.wt ) /

ggMarginal(g.rtl,groupFill = TRUE,margins = 'x')+ labs(tag="C") +

plot_layout(heights = c(1, 1.75)))

#sex-specific regressions

dat$scaled.log2tlmean<-scale(dat$log2tlmean)

rtl.lm.f<-lm(scaled.log2tlmean~scale(FA,scale=FALSE)+scale(Est.Age,scale=FALSE)+

population+Season_sampled+

scale(days_collection_to_assay,scale=FALSE)+

scale(days_since_calibrator_extracted,scale=FALSE),

#data=dat[dat$Est.Age<=max(data=dat[dat$sex=="M",]$Est.Age) & dat$sex=="F",])

data=dat[dat$sex=="F",])

Anova(rtl.lm.f,type="II")

summary(rtl.lm.f)

rtl.lm.m<-lm(scaled.log2tlmean~scale(FA,scale=FALSE)+scale(Est.Age,scale=FALSE)+

population+Season_sampled+

scale(days_collection_to_assay,scale=FALSE)+

scale(days_since_calibrator_extracted,scale=FALSE),

#data=dat[dat$Est.Age<8,])

data=dat[dat$sex=="M",])

Anova(rtl.lm.m,type="II")

summary(rtl.lm.m)

#sex-specific regression plots

g.f <- predict_response(rtl.lm.f, terms=c("Est.Age"),back_transform = FALSE,margin='empirical',ci_level = 0.95)

g1.f<-data.frame(g.f)

g.m <- predict_response(rtl.lm.m, terms=c("Est.Age"),back_transform = FALSE,margin='empirical',ci_level = 0.95)

g1.m<-data.frame(g.m)

#plot rTL across ages with predicted values from regression

g.rtl<-ggplot(dat,aes(x=Est.Age,y=log2tlmean,colour=sex),) +

geom_line(data=g1.m,aes(x=x,y=predicted),colour="#5090bf",size=1) +

geom_ribbon(data=g1.m,linetype='dotted',

aes(x=x,y=predicted,ymin=conf.low,ymax=conf.high),alpha=0.2,colour="#5090bf",fill="#5090bf")+

geom_rect(fill='#fcfbfa',aes(xmin=9.8,xmax=20,ymin=-2,ymax=2),colour='#fcfbfa')+

geom_line(data=g1.f,aes(x=x,y=predicted),colour="#fa8490",size=1) +

geom_ribbon(data=g1.f,linetype='dotted',

aes(x=x,y=predicted,ymin=conf.low,ymax=conf.high),alpha=0.2,colour="#fa8490",fill="#fa8490")+

geom_point(alpha=0.6)+

scale_x_continuous(breaks=seq(1,20,2))+labs(tag="C")+

labs(x = "Age",y = "log2 rTL",color = "Sex") +

theme_bw()+scale_colour_manual(values=c("#fa8490","#5090bf"))+

theme(panel.grid=element_blank(),legend.position='none',panel.background=element_rect(fill='#fcfbfa',colour='#aaaaaa'))

g.rtl

#analysis to test effects of measurement error (estimated age and rTL)

set.seed(1)

dat1<-dat

res.df<-data.frame(iter=c(1:10000),Bage=NA,Page=NA,

Bsex=NA,Psex=NA)

age.mae<-0.449

tel.mae<-median(abs(dat$rtl1-dat$rtl2), na.rm=TRUE)

for (iter in c(1:10000)) {

dat1$Est.Age.MAE<-dat1$Est.Age+runif(nrow(dat1),min = -age.mae,max = age.mae)

dat1$log2tlmean.rep<-log2(dat1$tlmean+runif(nrow(dat1),min = -tel.mae,max = tel.mae))

rtl.lm<-lm(scale(log2tlmean.rep)~scale(FA,scale=FALSE)+scale(Est.Age.MAE,scale=FALSE)+

sex+population+Season_sampled+

scale(days_collection_to_assay,scale=FALSE)+

scale(days_since_calibrator_extracted,scale=FALSE),

data=dat1)

res.df[iter,]$Bage<-summary(rtl.lm)$coefficients[3,1]

res.df[iter,]$Page<-Anova(rtl.lm,type="II")[2,4]

res.df[iter,]$Bsex<-summary(rtl.lm)$coefficients[4,1]

res.df[iter,]$Psex<-Anova(rtl.lm,type="II")[3,4]

}

#plot distributions of estimates and p-values

g.bage<-ggplot(res.df,aes(x=Bage))+geom_histogram()+theme_minimal()+xlab("Age estimate")+

geom_vline(xintercept=-0.051,colour='red',linetype='dashed')

g.page<-ggplot(res.df,aes(x=Page))+geom_histogram()+theme_minimal()+

geom_vline(xintercept=0.017,colour='red',linetype='dashed')+xlab("Age P-value")

g.bsex<-ggplot(res.df,aes(x=Bsex))+geom_histogram()+theme_minimal()+xlab("Sex estimate")+

geom_vline(xintercept=0.598,colour='red',linetype='dashed')

g.psex<-ggplot(res.df,aes(x=Psex))+geom_histogram()+theme_minimal()+

geom_vline(xintercept=0.003,colour='red',linetype='dashed')+xlab("Sex P-value")

ggsave("rTL_boostraps.png",plot=g.bage+g.page+g.bsex+g.psex,dpi=600,width=6,height=5)

#now do iterative subsampling to keep only females/males with similar ages

#this will test whether we see the same sex-differences in rTL after accounting for differences in age distribution

subsample.res.df<-data.frame(iter=c(1:10000),

Bsex=NA,Psex=NA,Pint=NA,wilcox.age.p=NA)

set.seed(1)

males<-dat[dat$sex=="M",]

females<-dat[dat$sex=="F",]

for(iter in c(1:10000)){

paired_data<-list()

if(iter %% 100==0){print(iter)}

subsample.res.df[iter,]$iter<-iter

paired_females<-logical(nrow(females))

for(i in 1:nrow(males)){

male<-males[i,]

#find females within 1 yr of age of ith male

f_cands<-females[abs(females$Est.Age-male$Est.Age)<=1 & !paired_females,]

#remove NAs. should be unnecessary

f_cands<-f_cands[!is.na(f_cands$band),]

#randomly select a female

if(nrow(f_cands)>0){

sampled_female<-f_cands[sample(c(1:nrow(f_cands)),1),]

female_index<-which(females$band==sampled_female$band)

paired_females[female_index]<-TRUE#make sure not to sample the same female again

paired_data[[i]]<-rbind(male[!is.na(male$band),],sampled_female[!is.na(sampled_female$band),])

paired_data_df<-do.call(rbind,paired_data)

}

}

subsample.res.df[iter,]$wilcox.age.p<-wilcox.test(paired_data_df$Est.Age~paired_data_df$sex,exact=FALSE)$p.value

wilcox.age.p<-subsample.res.df[iter,]$wilcox.age.p

#finished selecting samples, run analysis and store results

a1<-(lm(log2tlmean~scale(FA,scale=FALSE)+scale(Est.Age,scale=FALSE)+

sex+population+Season_sampled+

scale(days_collection_to_assay,scale=FALSE)+

scale(days_since_calibrator_extracted,scale=FALSE),data=paired_data_df))

subsample.res.df[iter,]$Bsex<-a1$coefficients["sexM"]

subsample.res.df[iter,]$Psex<-Anova(a1)[3,4]

a2<-lm(log2tlmean~scale(FA,scale=FALSE)+scale(Est.Age,scale=FALSE)*

sex+population+Season_sampled+

scale(days_collection_to_assay,scale=FALSE)+

scale(days_since_calibrator_extracted,scale=FALSE),data=paired_data_df)

subsample.res.df[iter,]$Pint<-Anova(a2,type="III")[9,4]

#}

}

paired_data_df$grp<-rep(1:31, each = 2)

ggplot(paired_data_df,aes())

nrow(subsample.res.df[subsample.res.df$Psex<0.05,])/10000#calc proportion of iterations with sig. sex differences in rTL

nrow(subsample.res.df[subsample.res.df$wilcox.age.p<0.05,])/10000#calc proportion of iterations with sig. sex differences in age

#plot histograms

g.bsex<-ggplot(subsample.res.df,aes(x=Bsex))+geom_histogram()+theme_minimal()+xlab("Estimate")+

geom_vline(xintercept=0.598,colour='blue',linetype='dashed')+

ggtitle("Sex estimate")+labs(tag="D")+

theme(panel.grid=element_blank(),legend.position='none',axis.text.y=element_blank(),axis.title.y=element_blank(),axis.ticks.x=element_line(),

panel.background=element_rect(fill='#fcfbfa',colour='black'),plot.title = element_text(size=8),axis.title = element_text(size=8))

g.psex<-ggplot(subsample.res.df,aes(x=Psex))+geom_histogram()+theme_minimal()+xlab("P-value")+

ggtitle("Sex P-value")+

geom_vline(xintercept=0.05,colour='red',linetype='dashed')+

theme(panel.grid=element_blank(),legend.position='none',axis.text.y=element_blank(),axis.title.y=element_blank(),axis.ticks.x=element_line(),

panel.background=element_rect(fill='#fcfbfa',colour='black'),plot.title = element_text(size=8),axis.title = element_text(size=8))

g.wsex<-ggplot(subsample.res.df,aes(x=wilcox.age.p))+geom_histogram()+theme_minimal()+xlab("P-value")+

ggtitle("Sex differences in age distribution")+

geom_vline(xintercept=0.05,colour='red',linetype='dashed')+

theme(panel.grid=element_blank(),legend.position='none',axis.text.y=element_blank(),axis.title.y=element_blank(),axis.ticks.x=element_line(),

panel.background=element_rect(fill='#fcfbfa',colour='black'),plot.title = element_text(size=8),axis.title = element_text(size=8))

ggsave('fig1_size_rtl_subsample1.svg',dpi=600,height=7.5,width=6,

plot=(g.fa | g.wt ) /

ggMarginal(g.rtl,groupFill = TRUE,margins = 'x') /

(g.bsex | g.psex | g.wsex) +

plot_layout(heights = c(1, 2, 0.5)))

sessionInfo()
