# Supplementary material for: Sex Differences in Telomere Length in a Bat With Female‐Biased Longevity
Source: Ecol Evol. 2025 May 14;15(5):e71378. doi: 10.1002/ece3.71378 (PMC12076057; doi:10.1002/ece3.71378)
Supplement: Supplementary file 3 — Appendix S3. Supporting Information. [file ECE3-15-e71378-s003.docx]

**Sex differences in telomere length in a bat with female-biased longevity**

Jack G. Rayner, Abigail Marshall, Danielle M. Adams, Jillian Kaiser, Katherine Armenta, Gerald S. Wilkinson

**Supporting Figures and Tables**


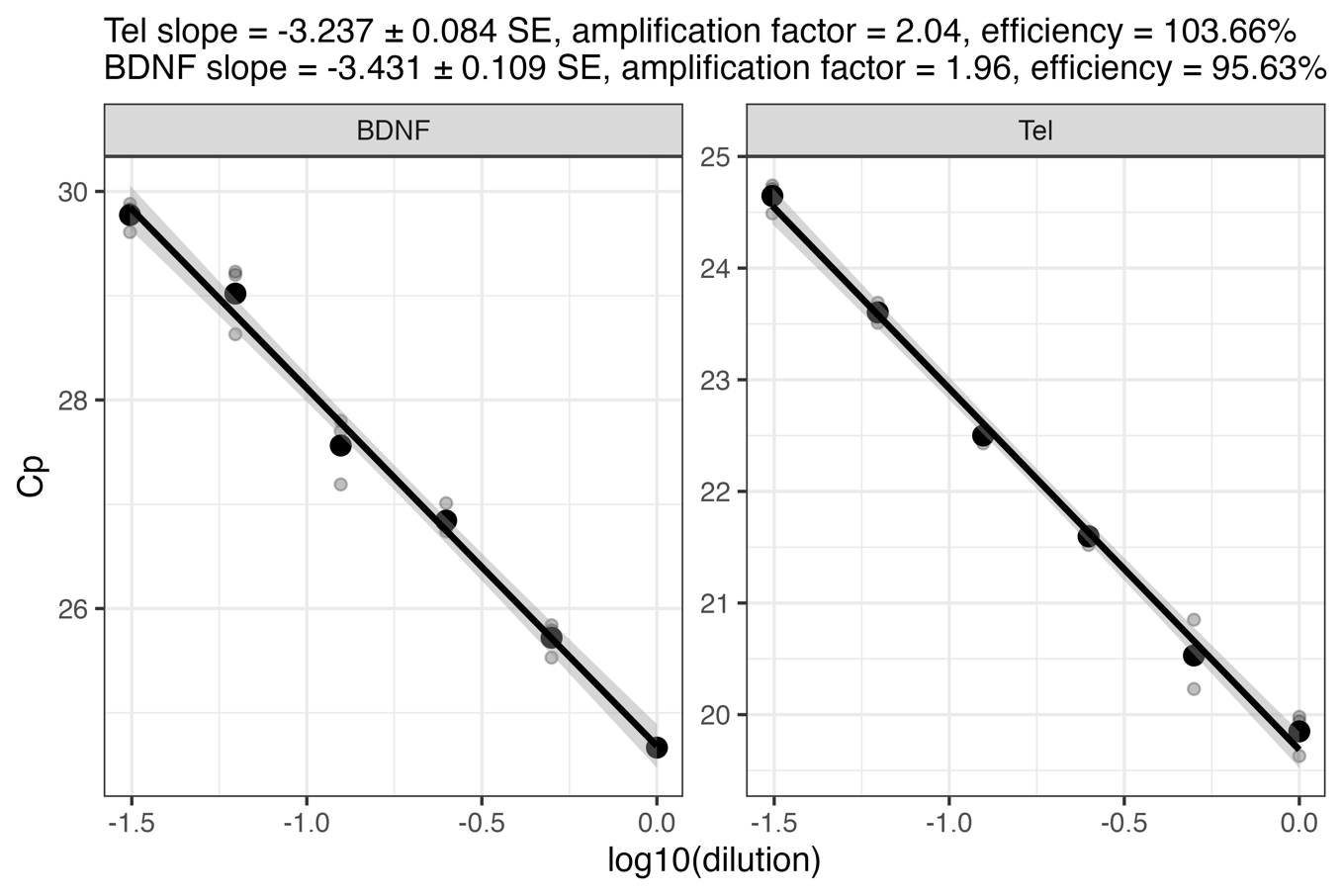


**Figure S1.** Amplification efficiencies for BDNF and Telomere primers. Larger points show means, smaller grey points show individual Cp values.


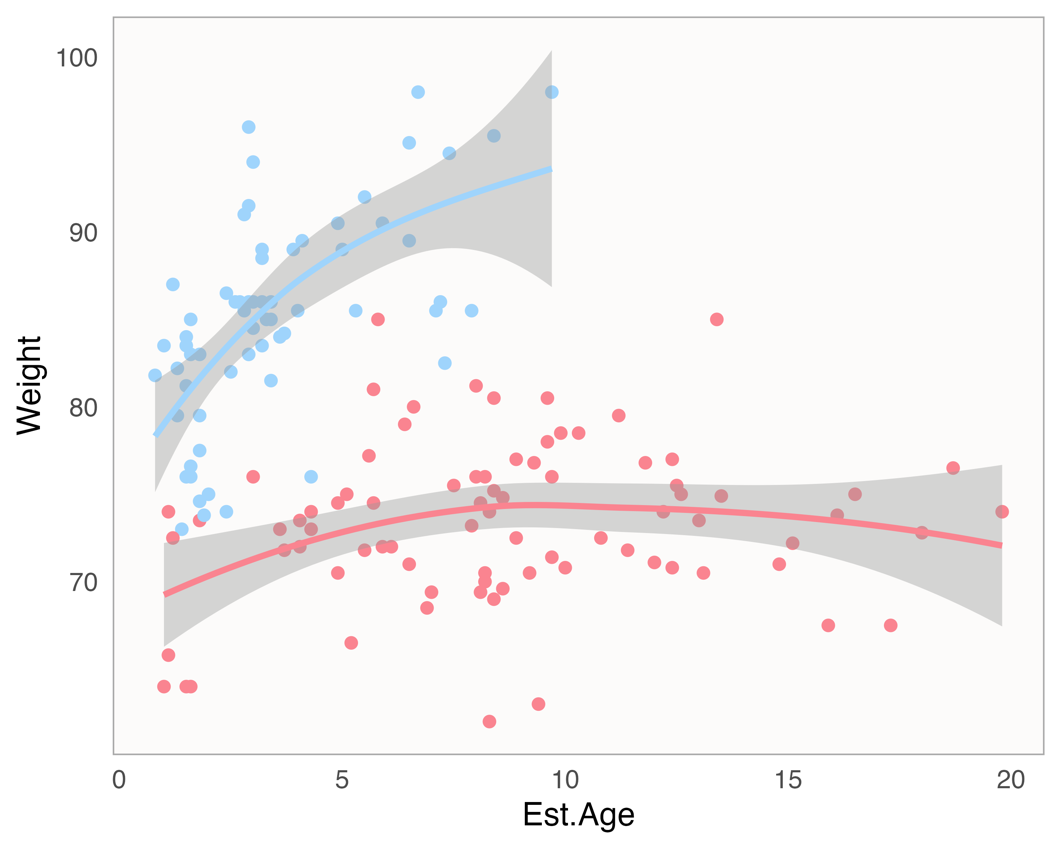


**Figure S2. Non-linear association between age and weight (z-scaled).** Lines show values from LOESS regression, with 95% confidence intervals. Males are indicated in blue, females in red.


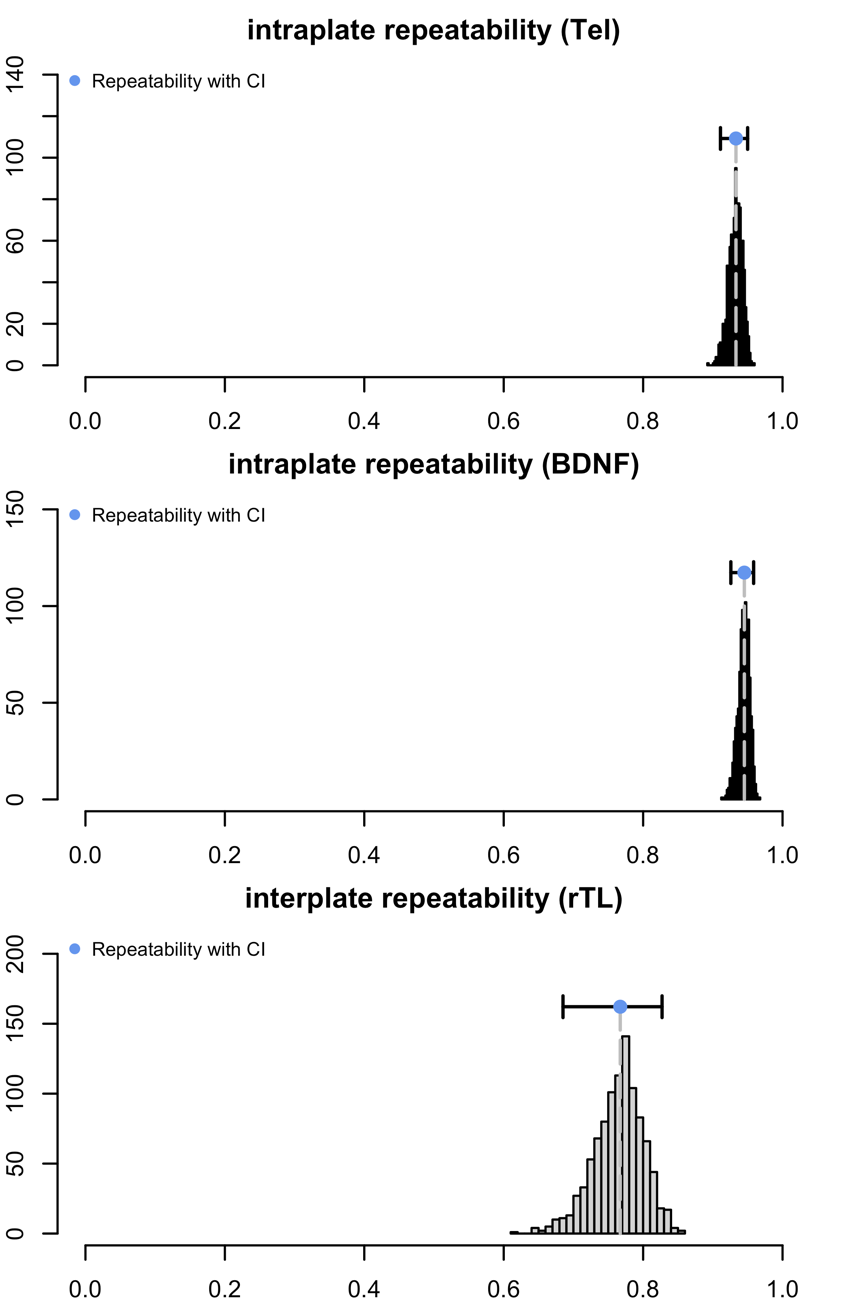


**Figure S3. Intra and interplate repeatabilities from 1000 bootstraps.** Repeatabilities are shown for triplicate crossing point values for telomere (Tel) and BDNF sequences, as well as interplate repeatability for rTL measures.


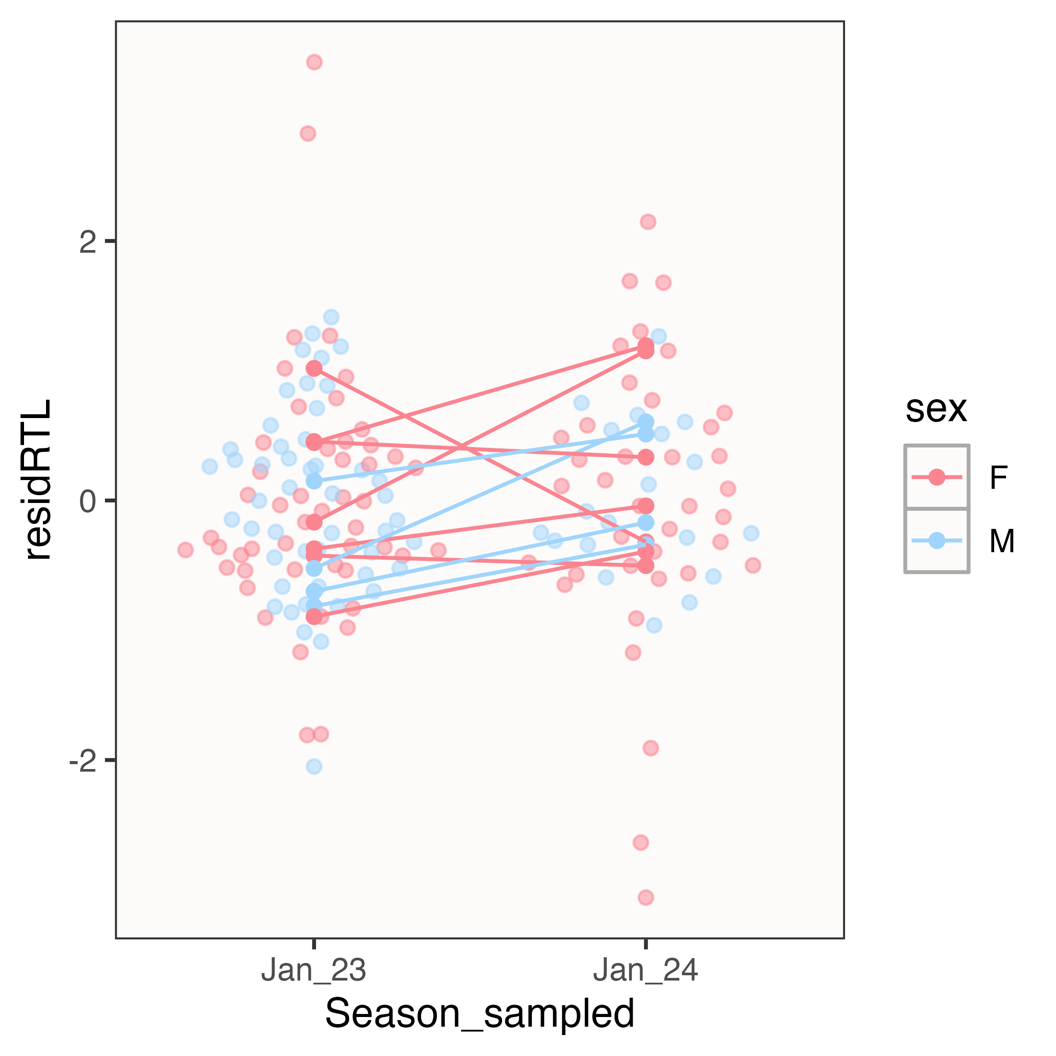


**Figure S4**. Residual rTL from repeated sampling of the same individuals (points joined by lines) between years, after running the analysis presented in Table 1 prior to the removal of samples from bats measured in both years. Residual values are shown to account for differences in mean rTL between years (Table 1).


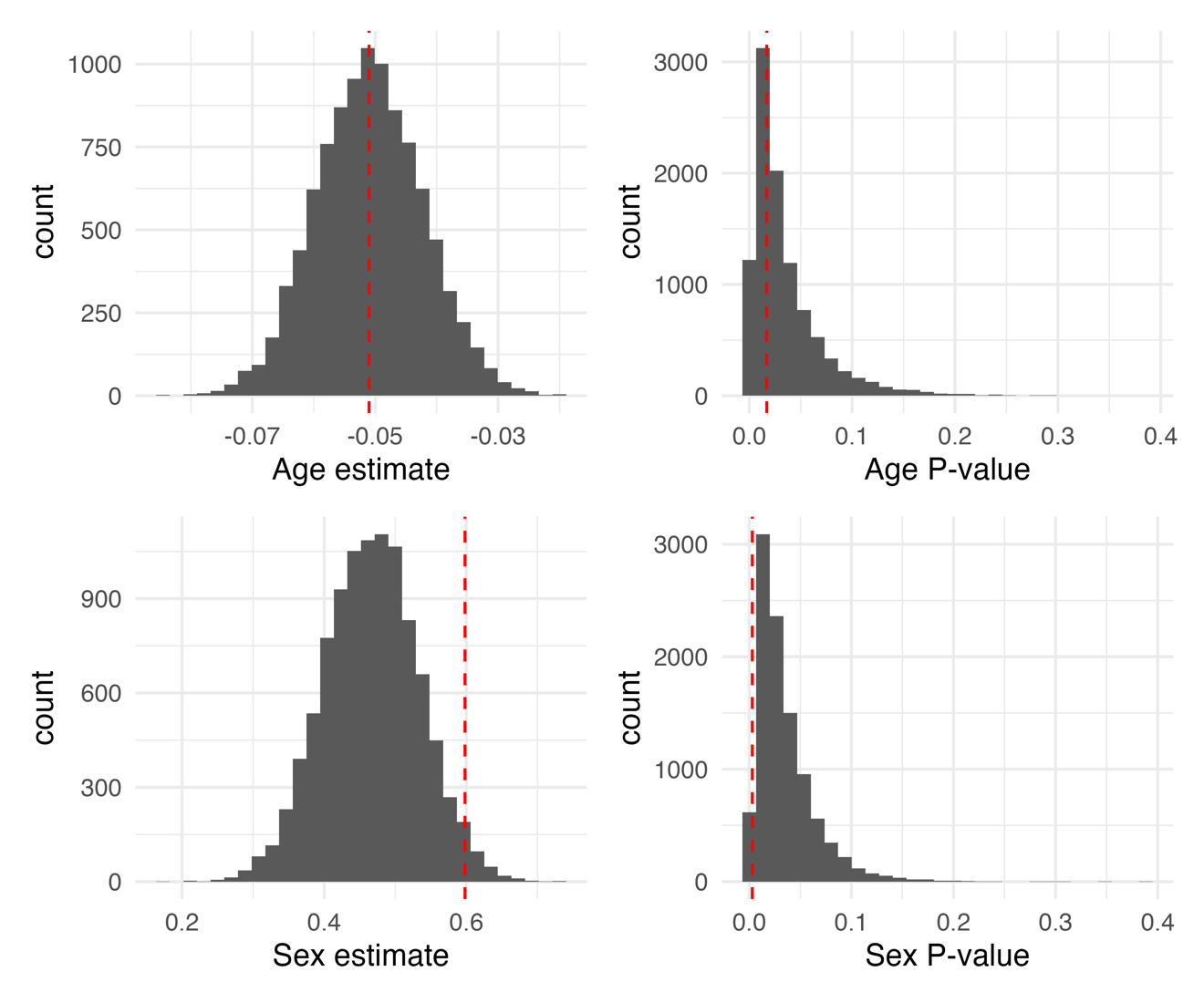


**Figure S5**. Estimates and P-values from linear models of rTL (see Table 1) for predictors of sex and age. In each of 10,000 bootstraps, we added a random value to age and rTL estimates with uniform distribution bounded between obs – MAE and obs + MAE.

**Table S1.** **Primer sequences**

| Primer | Sequence |
| --- | --- |
| BDNF F1 | 5’-AGCTGAGCGTGTGTGACAG-3’ |
| BDNF R1 | 5’-TGGGATTGCACTTGGTCTCGT-3’ |
| Tel1b | 5’-CGGTTTGTTTGGGTTTGGGTTTGGGTTTGGGTTTGGGTT -3’ |
| Tel2b | 5’-GGCTTGCCTTACCCTTACCCTTACCCTTACCCTTACCCT-3’ |

**Table S2. RT-qPCR mastermix conditions.** Thermocycler conditions were 5 minutes as 95c, following by 40 cycles of 15 seconds at 95C and one minute at 60C, followed by melt curve analysis.

|  | Tel | BDNF |
| --- | --- | --- |
| Sterile H_2_O | 6.2 | 5.27 |
| PowerUp™ SYBR™ Green Master Mix for qPCR | 10ul | 10ul |
| Primer1 (0.7uM) | 1.4ul | 1.4ul (BDNF F1) |
| Primer2 (0.7uM) | 1.4ul | 2.33ul (BDNF R1) |
| Sample (2ng/ul) | 1ul | 1ul |

**Table S3.** Results of linear regression of log_2_ mean rTL, z-transformed, after removal of the term for sample storage duration. The full model had an R^2^ of 0.198 (F_7,134_ = 4.730, P < 0.001).

| Predictor | Coef. ± SE | F | Df | P |
| --- | --- | --- | --- | --- |
| Intercept | -2.291 |  |  |  |
| Forearm length | -0.048 ± 0.049 | 0.968 | 1 | 0.327 |
| Est. age | -0.049 ± 0.023 | 4.872 | 1 | 0.029 |
| Sex (M) | 0.407 ± 0.208 | 3.818 | 1 | 0.053 |
| Population |  | 0.736 | 2 | 0.481 |
| *Caura* | 0.127 ± 0.220 |  |  |  |
| *Cumuto* | -0.288 ± 0.291 |  |  |  |
| Year sampled (Jan_2024) | 5.333 ± 2.187 | 5.946 | 1 | 0.016 |
| Calibrator sample storage duration | -0.016 ± 0.008 | 4.380 | 1 | 0.038 |
| Residuals | ± 0.919 |  | 134 |  |

**Table S4.** Results of linear regression of log_2_ mean rTL, z-transformed, after removal of the term for calibrator storage duration. The full model had an R^2^ of 0.213 (F_7,134_ = 5.179, P < 0.001).

| Predictor | Coef. ± SE | F | Df | P |
| --- | --- | --- | --- | --- |
| Intercept | 0.239 |  |  |  |
| Forearm length | -0.051 ± 0.049 | 1.095 | 1 | 0.247 |
| Est. age | -0.050 ± 0.022 | 5.111 | 1 | 0.025 |
| Sex (M) | 0.423 ± 0.207 | 4.199 | 1 | 0.042 |
| Population |  | 0.359 | 2 | 0.699 |
| *Caura* | 0.062 ± 0.214 |  |  |  |
| *Cumuto* | -0.224 ± 0.290 |  |  |  |
| Year sampled (Jan_2024) | -0.590 ± 0.554 | 1.135 | 1 | 0.289 |
| Sample storage duration | -0.019 ± 0.007 | 6.980 | 1 | 0.009 |
| Residuals | ± 0.910 |  | 134 |  |

**Table S5.** Results of linear regression of log_2_ mean rTL, z-transformed, after removal of collinear terms for year sampled and calibrator storage duration. The full model had an R^2^ of 0.206 (F_6,135_ = 5.847, P < 0.001).

| Predictor | Coef. ± SE | F | Df | P |
| --- | --- | --- | --- | --- |
| Intercept | -0.150 |  |  |  |
| Forearm length | -0.048 ± 0.049 | 0.962 | 1 | 0.328 |
| Est. age | -0.052 ± 0.022 | 5.258 | 1 | 0.023 |
| Sex (M) | 0.425 ± 0.207 | 4.229 | 1 | 0.042 |
| Population |  | 1.369 | 2 | 0.258 |
| *Caura* | 0.076 ± 0.213 |  |  |  |
| *Cumuto* | -0.385 ± 0.246 |  |  |  |
| Sample storage duration | -0.012 ± 0.003 | 20.985 | 1 | < 0.001 |
| Residuals | ± 0.911 |  | 135 |  |
